# Resveratrol Attenuates *Staphylococcus Aureus*-Induced Monocyte Adhesion through Downregulating PDGFR/AP-1 Activation in Human Lung Epithelial Cells

**DOI:** 10.3390/ijms19103058

**Published:** 2018-10-07

**Authors:** I-Ta Lee, Chih-Chung Lin, Chien-Chung Yang, Li-Der Hsiao, Ming-Yen Wu, Chuen-Mao Yang

**Affiliations:** 1 Department of Medical Research, Taichung Veterans General Hospital, Taichung 407, Taiwan; itl700128@gmail.com; 2 Department of Nursing, College of Nursing, Hungkuang University, Taichung 433, Taiwan; 3 Department of Anesthetics, Chang Gung Memorial Hospital at Linkuo and Chang Gung University, Kwei-San, Tao-Yuan 333, Taiwan; chihchung@adm.cgmh.org.tw (C.-C.L.); lidesiao@livemail.tw (L.-D.H.); 4 Department of Traditional Chinese Medicine, Chang Gung Memorial Hospital at Tao-Yuan, Kwei-San, Tao-Yuan 333, Taiwan; ita0128@gmail.com; 5 Department of Physiology and Pharmacology and Health Ageing Research Center, College of Medicine, Chang Gung University, Kwei-San, Tao-Yuan 333, Taiwan; it1700128@sunrise.hk.edu.tw; 6 Research Center for Chinese Herbal Medicine and Research Center for Food and Cosmetic Safety, College of Human Ecology, Chang Gung University of Science and Technology, Tao-Yuan 333, Taiwan

**Keywords:** lung inflammation, AP-1, resveratrol, *Staphylococcus aureus*, signaling pathways

## Abstract

*Staphylococcus aureus* (*S. aureus*) is a very common Gram-positive bacterium. It is widely distributed in air, soil, and water. *S. aureus* often causes septicemia and pneumonia in patients. In addition, it is considered to play a key role in mediating cell adhesion molecules upregulation. Resveratrol is a natural antioxidant with diverse biological effects, including the modulation of immune function, anti-inflammation, and cancer chemoprevention. In this study, we proved that *S. aureus*-upregulated vascular cell adhesion molecule-1 (VCAM-1) expression in human lung epithelial cells (HPAEpiCs) was inhibited by resveratrol. We also observed that resveratrol downregulated *S. aureus*-enhanced leukocyte count in bronchoalveolar lavage (BAL) fluid in mice. In HPAEpiCs, *S. aureus* stimulated c-Src, PDGFR, p38 MAPK, or JNK1/2 phosphorylation, which was inhibited by resveratrol. *S. aureus* induced the adhesion of THP-1 cells (a human monocytic cell line) to HPAEpiCs, which was also reduced by resveratrol. Finally, we found that *S. aureus* induced c-Src/PDGFR/p38 MAPK and JNK1/2-dependent c-Jun and ATF2 activation and in vivo binding of c-Jun and ATF2 to the VCAM-1 promoter, which were inhibited by resveratrol. Thus, resveratrol functions as a suppressor of *S. aureus*-induced inflammatory signaling, not only by inhibiting VCAM-1 expression but also by diminishing c-Src, PDGFR, JNK1/2, p38 MAPK, and AP-1 activation in HPAEpiCs.

## 1. Introduction

*Staphylococcus aureus* (*S. aureus*) is considered to be an important human pathogen, which often causes skin, respiratory, and urinary tract infections and bacteremia [1]. Lipoteichoic acid (LTA) is a cell wall component of Gram-positive bacteria that acts as an adhesion molecule to promote bacterial-cell binding and invasion [2]. In recent years, many studies have showed that LTA exhibits many of the inflammatory properties of lipopolysaccharide (LPS) and plays a key role in the inflammatory responses enhanced by Gram-positive bacterial infection [3]. Toll-like receptors (TLRs) recognize microbial components via different signaling cascades and cause multiple responses in various cells [3]. *S. aureus* is recognized by TLR2, which induces innate immune responses by activating many protein kinases, inflammatory cytokines and chemokines, or transcription factors [4].

Many studies have indicated that lung inflammation is regulated by interactions between the vascular endothelium and circulating polymorphonuclear cells (PMNs). Previous studies have shown that the induction of adhesion molecules on the cell surface plays a very important role in the inflammatory responses [5]. Lazaar et al. proved that patients with respiratory diseases have upregulated adhesion activity associated with adhesion molecules between PMNs and epithelial cells [6]. The upregulation of adhesion molecules causes the recruitment of PMNs to the areas of inflammatory tissue. Adhesion molecules can be mainly divided into four types including cadherins, integrins, selectins, and immunoglobulin (Ig)-related cell adhesion molecules (CAMs) [7]. Vascular cell adhesion molecule-1 (VCAM-1) is a cell surface glycoprotein that regulates the adhesion of PMNs, thereby promoting the migration of PMNs across the vascular endothelium barrier and then interacting with lung epithelial cells [8]. Tumor necrosis factor (TNF)-α or LPS has been shown to upregulate VCAM-1 expression on the surface of various cell types [5,8]. Therefore, to elucidate the mechanisms by which *S. aureus* induces VCAM-1 expression is considered a new method for treating respiratory diseases. LPS has been shown to enhance VCAM-1 expression via the TLR4/myeloid differentiation primary response 88 (MyD88)/c-Src/p38 mitogen-activated protein kinase (MAPK)-dependent activating transcription factor 2 (ATF2) pathway [9]. In addition, platelet-derived growth factor receptor (PDGFR), MAPKs, and activator protein-1 (AP-1) have been shown to regulate VCAM-1 induction [10,11]. Thus, we investigated whether *S. aureus* could stimulate VCAM-1 expression via these signaling components in human pulmonary alveolar epithelial cells (HPAEpiCs) and the lungs of mice. 

Polyphenols have been shown to decrease inflammatory responses [8]. Resveratrol (trans-3,4’,5-trihydroxystilbene) is a natural polyphenolic compound commonly found in berries, grapes, and peanuts. In addition, a previous study proved that resveratrol could inhibit inflammation via the inhibition of nuclear factor (NF)-κB and AP-1 [12]. Kim et al. indicated that resveratrol suppresses the transcription of matrix metalloproteinase-9 (MMP-9) by the inhibition of both NF-κB and AP-1 transactivation [13]. Interestingly, resveratrol similarly regulates MAPKs activation in a dose-dependent manner [14,15,16]. Donnelly et al. proved that resveratrol could exert anti-inflammatory activity in epithelial cells [17]. These findings suggest that resveratrol might be useful as an anti-inflammatory modulator of lung inflammation. Thus, we determined whether resveratrol could inhibit VCAM-1 expression induced by *S. aureus* via the reduction of various inflammatory signaling molecules. We reported here for the first time that in HPAEpiCs, resveratrol reduced *S. aureus*-induced VCAM-1 expression via the inhibition of c-Src, PDGFR, c-Jun N-terminal kinase (JNK)1/2, p38 MAPK, and AP-1 activation.

## 2. Results

### 2.1. S. aureus Induces VCAM-1 Expression in HPAEpiCs via c-Src

**Figure 1:**
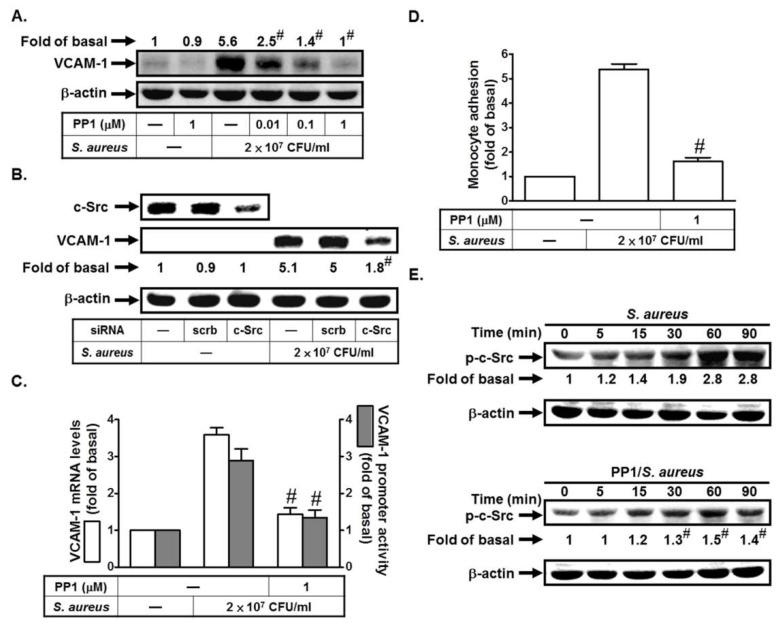
*S. aureus* induces vascular cell adhesion molecule-1 (VCAM-1) expression in human lung epithelial cells (HPAEpiCs) via c-Src. (**A**) Cells were pretreated with PP1 for 1 h, and then treated with *S. aureus* for 24 h. The protein levels of VCAM-1 were determined by Western blot. (**B**) Cells were transfected with scrambled or c-Src siRNA, and then incubated with *S. aureus* for 24 h. The protein levels of c-Src and VCAM-1 were determined by Western blot. (**C**,**D**) Cells were pretreated with PP1 for 1 h, and then treated with *S. aureus* for 4 h, 6 h, or 24 h. The mRNA levels and promoter activity of VCAM-1 were determined by real-time PCR and promoter activity (**C**). The THP-1 cells (a human monocytic cell line) adherence was measured (**D**). (**E**) Cells were pretreated without or with PP1 for 1 h, and then incubated with *S. aureus* for the indicated times. The expression of phospho-c-Src was determined by Western blot. *n* = 3–4, # *p* < 0.01, as compared with the cells exposed to *S. aureus* alone (**A**,**C**,**D**,**E**) or scrambled siRNA + *S. aureus* (**B**).

c-Src has been shown to be involved in VCAM-1 upregulation induced by LPS [9]. Here, we investigated whether *S. aureus* could stimulate VCAM-1 expression via c-Src in HPAEpiCs. As shown in Figure 1A, *S. aureus* induced VCAM-1 protein levels, which was inhibited by PP1 (an inhibitor of c-Src). To further confirm the role of c-Src in *S. aureus*-induced VCAM-1 expression, the siRNA of c-Src was used. As shown in Figure 1B, transfection with c-Src siRNA markedly reduced *S. aureus*-induced VCAM-1 expression. In addition, *S. aureus*-induced VCAM-1 mRNA levels and promoter activity and monocyte adhesion were also reduced by PP1 (Figure 1C,D). Moreover, we found that *S. aureus* could induce c-Src phosphorylation in a time-dependent manner, which was reduced by preincubation with PP1 (Figure 1E). Taken together, we suggest that *S. aureus* induces VCAM-1 expression via a c-Src signaling pathway.

### 2.2. S. aureus Induces VCAM-1 Expression in HPAEpiCs via PDGFR

**Figure 2:**
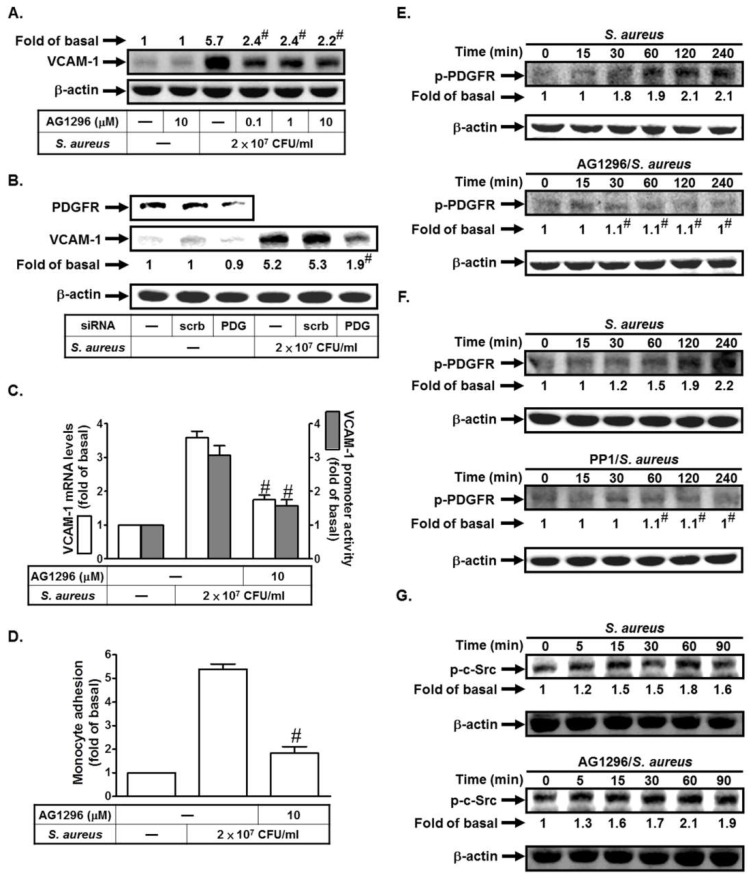
*S. aureus* induces VCAM-1 expression in HPAEpiCs via PDGFR. (**A**) Cells were pretreated with AG1296 for 1 h, and then treated with *S. aureus* for 24 h. The protein levels of VCAM-1 were determined. (**B**) Cells were transfected with scrambled or PDGFR siRNA, and then incubated with *S. aureus* for 24 h. The protein levels of PDGFR and VCAM-1 were determined. (**C**,**D**) Cells were pretreated with AG1296 for 1 h, and then treated with *S. aureus* for 4, 6, or 24 h. The mRNA levels and promoter activity of VCAM-1 were determined (**C**). The THP-1 cells adherence was measured (**D**). (**E**,**F**) Cells were pretreated without or with AG1296 or PP1 for 1 h, and then incubated with *S. aureus* for the indicated times. The expression of phospho-PDGFR was determined by Western blot. (**G**) Cells were pretreated without or with AG1296 for 1 h, and then incubated with *S. aureus* for the indicated times. The expression of phospho-c-Src was determined by Western blot. *n* = 3–4, # *p* < 0.01, as compared with the cells exposed to *S. aureus* alone (**A**,**C**,**D,E,F**) or scrambled siRNA + *S. aureus* (**B**).

We previously indicated that enterovirus 71 (EV71) could stimulate PDGFR-dependent VCAM-1 expression in vascular smooth muscle cells [11]. Here, we investigated whether *S. aureus* could stimulate VCAM-1 expression via PDGFR in HPAEpiCs. As shown in Figure 2A, *S. aureus* induced VCAM-1 protein levels, which was inhibited by AG1296 (an inhibitor of PDGFR). To further confirm the role of PDGFR in *S. aureus*-induced VCAM-1 expression, the siRNA of PDGFR was used. As shown in Figure 2B, transfection with PDGFR siRNA markedly reduced *S. aureus*-induced VCAM-1 expression. In addition, *S. aureus*-induced VCAM-1 mRNA levels and promoter activity and monocyte adhesion were also reduced by AG1296 (Figure 2C,D). Moreover, we found that *S. aureus* could induce PDGFR phosphorylation in a time-dependent manner, which was reduced by preincubation with AG1296 (Figure 2E). c-Src has been shown to mediate PDGFR activation in response to various stimuli [18,19,20]. Thus, we investigated the relationship between c-Src and PDGFR in *S. aureus*-stimulated HPAEpiCs. As shown in Figure 2F, pretreatment with PP1 inhibited *S. aureus*-induced PDGFR phosphorylation. However, AG1296 had no effects on *S. aureus*-induced c-Src activation (Figure 2G). Thus, we suggest that *S. aureus* induces VCAM-1 expression via c-Src-dependent PDGFR activation. 

### 2.3. S. aureus Induces VCAM-1 Expression in HPAEpiCs via MAPKs

**Figure 3:**
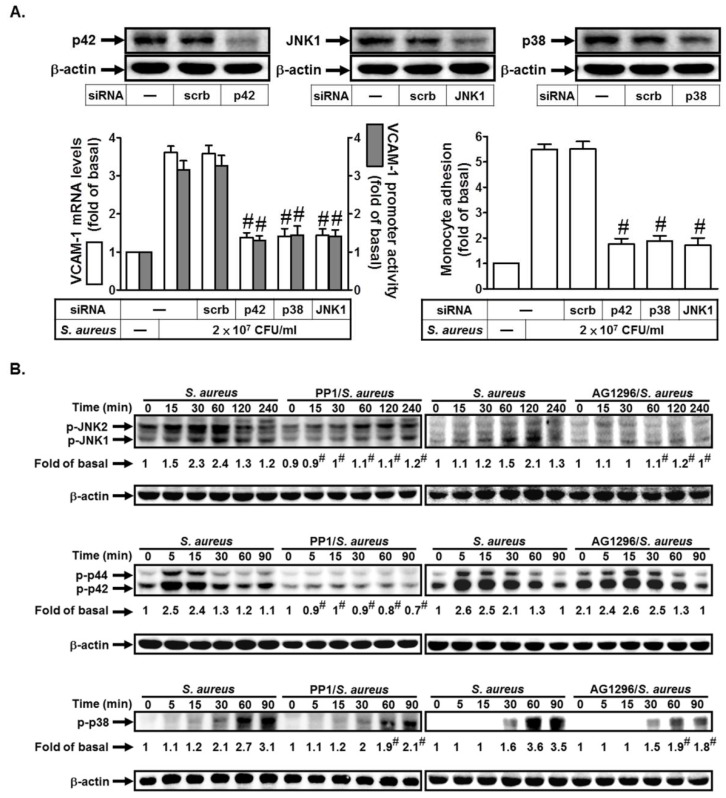
*S. aureus* induces VCAM-1 expression in HPAEpiCs via PDGFR. (**A**) Cells were transfected with scrambled, p42, p38, or JNK1 siRNA, and then incubated with *S. aureus* for 4 h, 6 h, or 24 h. The mRNA levels and promoter activity of VCAM-1 and the THP-1 cells adherence were measured. (**B**) Cells were pretreated without or with PP1 or AG1296 for 1 h, and then incubated with *S. aureus* for the indicated times. The expression of phospho-p38 MAPK, phospho-p42/p44 MAPK, and phospho-JNK1/2 were determined by Western blot. *n* = 3–4, # *p* < 0.01, as compared with the cells exposed to *S. aureus* + scrambled siRNA (**A**) or *S. aureus* alone (**B**).

Previous study indicated that cytokines, neurotransmitters, growth factors, and chemical and mechanical stressors could induce MAPKs activation [5]. Here, we also investigated whether MAPKs were involved in *S. aureus*-induced VCAM-1 expression. As shown in Figure 3A, transfection with siRNA of p42, JNK1, or p38 markedly inhibited *S. aureus*-induced VCAM-1 mRNA levels and promoter activity and monocyte adhesion. Finally, we investigated the relationship of c-Src, PDGFR, and MAPKs in *S. aureus*-treated HPAEpiCs. As shown in Figure 3B, pretreatment with PP1 markedly inhibited *S. aureus*-induced JNK1/2, p42/p44 MAPK, and p38 MAPK activation. However, AG1296 had an inhibitory effect on p38 MAPK or JNK1/2, but not p42/p44 MAPK activation induced by *S. aureus* (Figure 3B). Therefore, *S. aureus* induces VCAM-1 expression via c-Src/PDGFR-dependent p38 MAPK and JNK1/2 activation in HPAEpiCs.

### 2.4. S. aureus Induces VCAM-1 Expression in HPAEpiCs via AP-1

**Figure 4:**
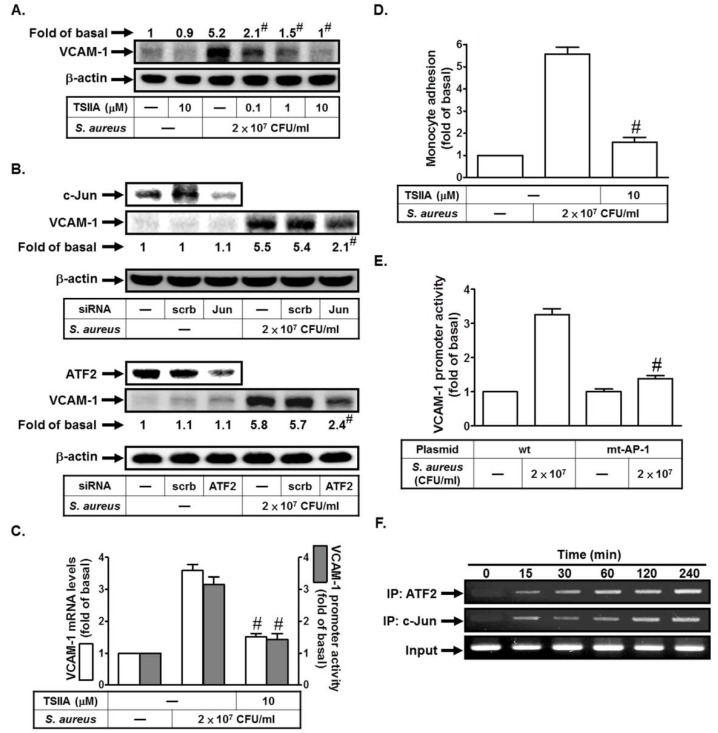
*S. aureus* induces VCAM-1 expression via AP-1. (**A**) Cells were pretreated with Tanshinone IIA for 1 h, and then treated with *S. aureus* for 24 h. The protein levels of VCAM-1 were determined. (**B**) Cells were transfected with scrambled, c-Jun, or ATF2 siRNA, and then incubated with *S. aureus* for 24 h. The protein levels of c-Jun, ATF2, and VCAM-1 were determined. (**C**,**D**) Cells were pretreated with Tanshinone IIA for 1 h, and then treated with *S. aureus* for 4 h, 6 h, or 24 h. The mRNA levels and promoter activity of VCAM-1 were determined (**C**). The THP-1 cells adherence was measured (**D**). (**E**) Cells were transfected with wild-type VCAM-1 promoter and AP-1-mutated VCAM-1 promoter, and then incubated with *S. aureus* for 6 h. The promoter activity of VCAM-1 was determined. (**F**) Cells were treated with *S. aureus* for the indicated times. ATF2 and c-Jun binding activities were analyzed by a ChIP assay. *n* = 3–4, # *p* < 0.01, as compared with the cells exposed to *S. aureus* alone (**A,C,D**) or *S. aureus* + scrambled siRNA (**B**). # *p* < 0.01, as compared with the cells transfected with wild-type VCAM-1 promoter stimulated by *S. aureus* (**E**).

Previous study proved that bacterial infections could enhance AP-1 activity [5]. AP-1 is composed of proteins belonging to the c-Fos, c-Jun, ATF, and JDP families, which can regulate gene expression in response to various stimuli [5]. Here, we investigated whether *S. aureus* could stimulate VCAM-1 expression via AP-1 in HPAEpiCs. As shown in Figure 4A, *S. aureus* induced VCAM-1 protein levels, which was inhibited by Tanshinone IIA (an inhibitor of AP-1). To further confirm the role of AP-1 in *S. aureus*-induced VCAM-1 expression, the siRNA of c-Jun or ATF2 was used. As shown in Figure 4B, transfection with c-Jun or ATF2 siRNA markedly reduced *S. aureus*-induced VCAM-1 expression. In addition, *S. aureus*-induced VCAM-1 mRNA levels and promoter activity and monocyte adhesion were also reduced by Tanshinone IIA (Figure 4C,D). On the other hand, we found that *S. aureus*-induced VCAM-1 promoter activity was prominently lost in HPAEpiCs transfected with the point-mutated AP-1 VCAM-1 promoter (Figure 4E). Finally, we investigated whether *S. aureus*-induced recruitment of c-Jun or ATF2 to VCAM-1 promoter was involved in VCAM-1 gene expression. We proved that *S. aureus* stimulated in vivo binding of ATF2 or c-Jun to the VCAM-1 promoter in a time-dependent manner in these cells (Figure 4F). Taken together, we suggest that *S. aureus* induces VCAM-1 expression via an AP-1 signaling pathway.

### 2.5. S. aureus Induces AP-1 Activation via c-Src/PDGFR/JNK1/2 and p38 MAPK Pathways 

**Figure 5:**
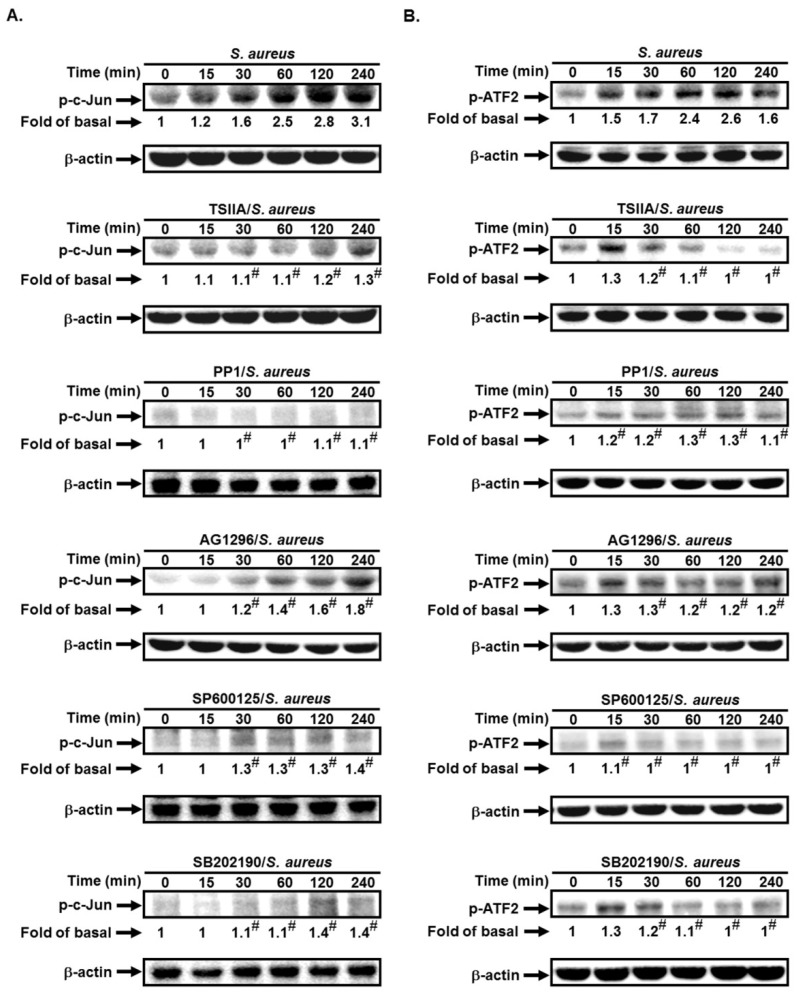
*S. aureus* induces AP-1 activation via c-Src/PDGFR/JNK1/2 and p38 MAPK pathways. Cells were pretreated without or with PP1, AG1296, SP600125, or SB202190 for 1 h, and then incubated with *S. aureus* for the indicated times. The expression of (**A**) phospho-c-Jun and (**B**) phospho-ATF2 was determined by Western blot. *n* = 3–4, # *p* < 0.01, as compared with the cells exposed to *S. aureus* alone.

AP-1 has been shown to be mediated through various signaling pathways, such as c-Src, PDGFR, PI3K/Akt, or MAPKs [19,20]. Here, we investigated whether c-Src, PDGFR, JNK1/2, and p38 MAPK were involved in *S. aureus*-induced c-Jun and ATF2 activation. As shown in Figure 5A,B, pretreatment with PP1, AG1296, SP600125, or SB202190 markedly reduced *S. aureus*-induced c-Jun and ATF2 phosphorylation in these cells. Thus, we suggest that *S. aureus* induces AP-1 activation via c-Src/PDGFR-dependent JNK1/2 and p38 MAPK phosphorylation. 

### 2.6. Resveratrol Inhibits S. aureus-Induced VCAM-1 Expression and Inflammatory Signaling Pathways

**Figure 6:**
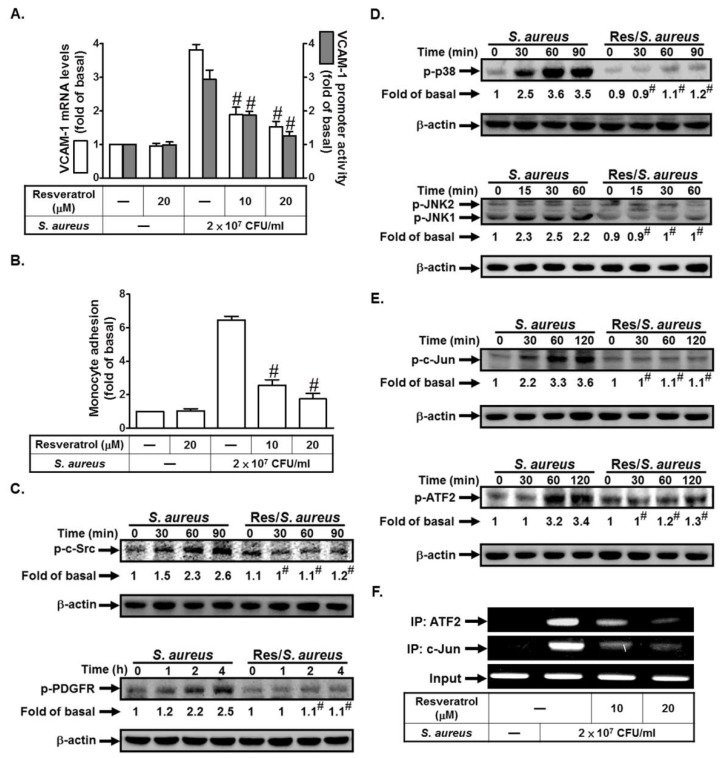
Resveratrol inhibits *S. aureus*-induced VCAM-1 expression and inflammatory signaling pathways. (**A**) Cells were pretreated with resveratrol for 24 h, and then incubated with *S. aureus* for 4 h or 6 h. The mRNA levels and promoter activity of VCAM-1 were determined. (**B**) Cells were pretreated with resveratrol for 24 h, and then incubated with *S. aureus* for 24 h. The THP-1 cells adherence was measured. (**C**–**E**) Cells were pretreated without or with resveratrol for 24 h, and then incubated with *S. aureus* for the indicated times. The expression of phospho-c-Src, phospho-PDGFR, phospho-p38 MAPK, phospho-JNK1/2, phospho-c-Jun, and phospho-ATF2 were determined by Western blot. (**F**) Cells were pretreated with resveratrol for 24 h, and then incubated with *S. aureus* for 4 h. ATF2 and c-Jun binding activities were analyzed by a ChIP assay. *n* = 3–4, # *p* < 0.01, as compared with the cells exposed to *S. aureus* alone.

Resveratrol is an antioxidant-like compound found in red wine, berries, and peanuts. It has been shown to inhibit inflammation [8]. First, we investigated whether resveratrol could inhibit *S. aureus*-induced VCAM-1 expression. As shown in Figure 6A, pretreatment with resveratrol (10 or 20 μM) markedly inhibited *S. aureus*-induced VCAM-1 mRNA levels and promoter activity. *S. aureus*-induced monocyte adhesion was decreased by pretreatment with resveratrol (10 or 20 μM) (Figure 6B). We further investigated whether resveratrol could inhibit VCAM-1 expression via the inhibition of the inflammatory signaling pathways. We found that resveratrol had an inhibitory effect on *S. aureus*-induced c-Src, PDGFR, p38 MAPK, JNK1/2, c-Jun, and ATF2 phosphorylation (Figure 6C–E). Finally, we demonstrated that resveratrol pretreatment markedly reduced *S. aureus*-induced in vivo binding of ATF2 or c-Jun to the VCAM-1 promoter (Figure 6F). Taken together, these data suggest that resveratrol can inhibit VCAM-1 expression via the inhibition of the c-Src/PDGFR/JNK1/2 and p38 MAPK/AP-1 pathways.

### 2.7. Resveratrol Inhibits S. aureus-Induced VCAM-1 Expression in the Lungs of Mice

**Figure 7:**
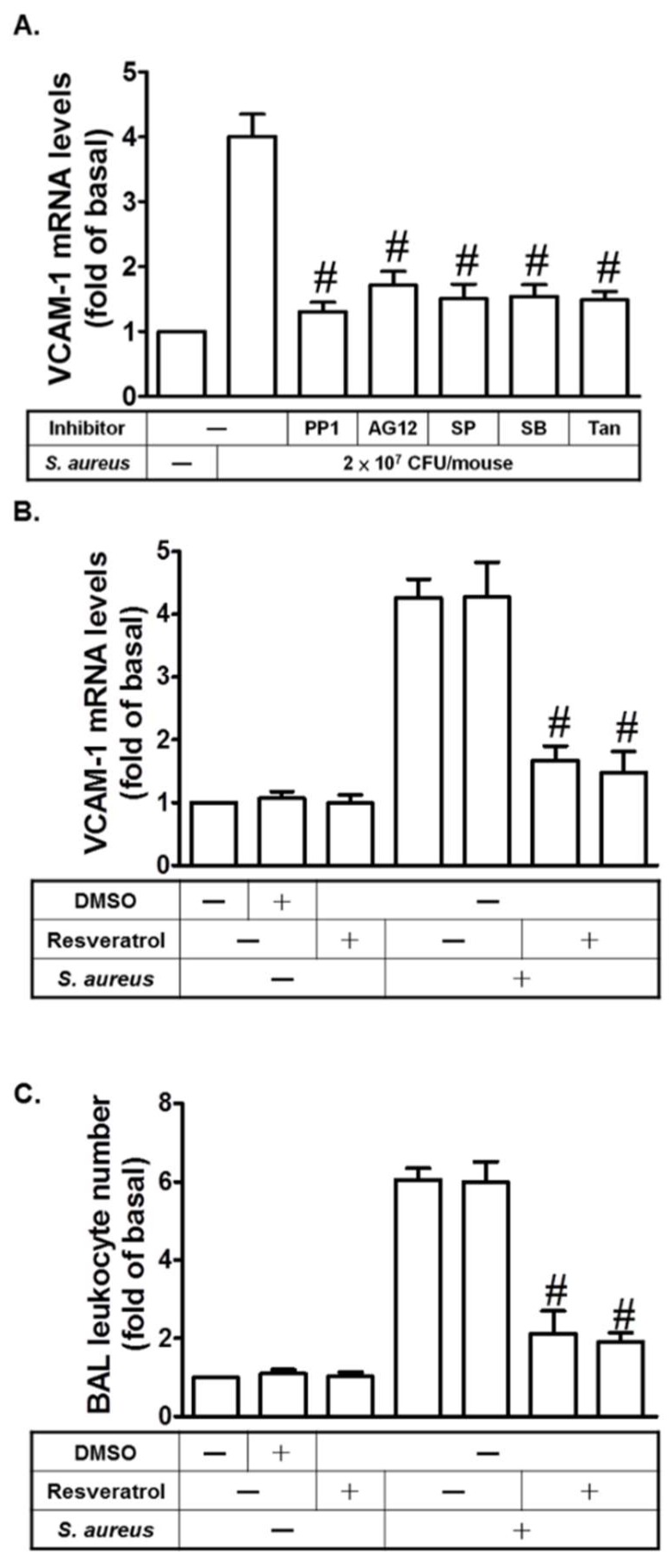
Resveratrol inhibits *S. aureus*-induced VCAM-1 expression in the lungs of mice. (**A**) Mice were intraperitoneal given one dose of PP1, AG1296, SP600125, SB202190, or Tanshinone IIA for 2 h prior to *S. aureus* (2 × 10^7^ CFU/mouse) treatment, and sacrificed after 24 h. Preparation of lung tissues was analyzed by real-time PCR to determine the levels of VCAM-1 mRNA. (**B**) Mice were intraperitoneal given one dose of resveratrol for 24 h prior to *S. aureus* (2 × 10^7^ CFU/mouse) treatment, and sacrificed after 24 h. Preparation of lung tissues was analyzed by real-time PCR to determine the levels of VCAM-1 mRNA. (**C**) Mice were intraperitoneal given one dose of resveratrol for 24 h prior to *S. aureus* (2 × 10^7^ CFU/mouse) treatment, and sacrificed after 24 h. Bronchoalveolar lavage (BAL) fluid was acquired and leukocyte count was determined. *n* = 4–5, # *p* < 0.01, as compared with the mice exposed to *S. aureus* alone.

In an in vivo study, mice were intra-tracheally administered with *S. aureus*. As shown in Figure 7A, *S. aureus* induced VCAM-1 mRNA expression in the lungs of mice, which was reduced by PP1, AG1296, SP600125, SB202190, or Tanshinone IIA. On the other hand, we showed that resveratrol could inhibit *S. aureus*-induced VCAM-1 mRNA expression in the lungs of mice (Figure 7B). Pretreatment with resveratrol could inhibit *S. aureus*-induced leukocyte count in bronchoalveolar lavage (BAL) fluid in mice (Figure 7C). Thus, we also demonstrated that resveratrol can inhibit lung inflammation via the inhibition of these inflammatory molecules.

## 3. Discussion

*S. aureus* has been implicated in the pathophysiology of many inflammatory lung diseases, including septicemia or pneumonia [1,2]. Lazaar et al. proved that patients with respiratory diseases have an upregulated adhesion activity associated with adhesion molecules between PMNs and epithelial cells [6]. It was found that the upregulation of cell adhesion molecules and proinflammatory cytokines plays a key role in mediating *S. aureus*-induced inflammation. Moreover, resveratrol has been proven to be effective in suppressing inflammation [21]. Resveratrol has been shown to inhibit the expression of cell adhesion molecules [22]. However, the molecular mechanisms underlying the resveratrol-inhibited expression of adhesion molecules in HPAEpiCs remain unclear. Here, we proved that *S. aureus* induced c-Src/PDGFR/JNK1/2 and p38 MAPK-dependent AP-1 activation and VCAM-1 expression in HPAEpiCs. In an in vivo study, *S. aureus* enhanced leukocyte count in the BAL fluid of mice. Pretreatment with resveratrol inhibited *S. aureus*-induced VCAM-1 expression, THP-1 cell (a human monocytic cell line) adherence, and the activation of c-Src, PDGFR, JNK1/2, p38 MAPK, c-Jun, and ATF2. The current study suggests that resveratrol exerted a protective effect on lung inflammation by the inhibition of VCAM-1 expression in lung tissues.

*S. aureus* has been proven to be a major pathogen of community-acquired and nosocomial infections. The main site of infection is usually a breach in the skin, which can cause skin and wound infections, but *S. aureus* can also infect any tissue of the body, leading to various life-threatening diseases [23]. The invasion of pathogens promotes the production and release of proinflammatory cytokines [24]. TLRs recognize microbial components via different signaling cascades and cause multiple responses [3]. *S. aureus* is recognized by TLR2, which induces innate immune responses by activating many protein kinases, inflammatory cytokines and chemokines, or transcription factors [4]. Indeed, we also found that transfection with siRNA of TLR2, but not TLR4, inhibited *S. aureus*-induced VCAM-1 mRNA levels (data not shown). Thus, we suggest that TLR2 is involved in *S. aureus*-induced inflammatory signaling pathways.

Resveratrol is a polyphenol nonflavonoid compound present in strongly pigmented vegetables and fruits [25]. Many studies indicated that resveratrol inhibits inflammatory response via the inhibition of the initial recruitment of PMN and the production of proinflammatory mediators [26]. A previous study indicated that resveratrol inhibited ICAM-1 and VCAM-1 expression and neutrophil adhesion induced by TNF-α [27]. Moreover, we observed that, in HPAEpiCs, resveratrol can reduce *S. aureus*-induced VCAM-1 expression and monocyte adhesion. BAL provides a very important and practical diagnostic tool for the diagnosis of a variety of chronic lung diseases. By analyzing BAL fluid, we can determine white blood cell (WBC) profiles as well as detect respiratory pathogens [7]. We found that resveratrol also decreased *S. aureus*-induced leukocyte counts in the BAL fluid in mice. These data show that resveratrol might be useful as an anti-inflammatory modulator of lung inflammation.

Src family kinases (SFKs) have been shown to mediate various cellular processes including survival, migration, and proliferation. Numerous studies have implicated c-Src as a regulator of TLR signaling [3,28]. In our study, we demonstrated that the inhibition of c-Src reduced *S. aureus*-induced inflammatory responses. Moreover, resveratrol had an inhibitory effect on *S. aureus*-induced c-Src activation in HPAEpiCs. Tyrosine kinases receptors have been shown to regulate tissue repair and normal cellular homeostasis. Abnormal signaling patterns of these receptors are associated with the development of many diseases [5]. The EGFR and PDGFR families are the two major families of tyrosine kinase receptors. In recent years, they have been shown to play very important roles in potential therapeutic targets for respiratory diseases. In addition, these receptors have been shown to play critical roles in tissue remodeling in bronchitis and pulmonary fibrosis. Previous studies proved that c-Src could regulate cytokine-induced PDGFR transactivation through PDGFR phosphorylation [19,20]. In our study, we demonstrated that *S. aureus* induced c-Src-dependent PDGFR activation in HPAEpiCs. In addition, PDGFR activation also plays a critical role in mediating VCAM-1 expression induced by *S. aureus*. MAPKs are important for intracellular signaling and play critical roles in regulating inflammatory responses [2]. In this study, we found that *S. aureus*-induced VCAM-1 expression and monocyte adhesion were inhibited by pretreatment with the inhibitors of MAPKs. Although c-Src or PDGFR activation has been shown to mediate the activation of all three MAPKs in response to various stimuli [11,19,20], we only found that the inhibition of c-Src reduced p42/p44 MAPK, JNK1/2, and p38 MAPK activation induced by *S. aureus*. However, PDGFR inhibition only reduced JNK1/2 and p38 MAPK phosphorylation induced by *S. aureus*. Thus, we considered that although p42/p44 MAPK plays a key role in regulating VCAM-1 expression induced by *S. aureus*, it is not mediated via a c-Src/PDGFR-dependent pathway in these cells. In the future, we will investigate the mechanisms of *S. aureus*-mediated p42/p44 MAPK activation in HPAEpiCs. Moreover, pretreatment with resveratrol markedly reduced *S. aureus*-mediated p38 MAPK and JNK1/2 phosphorylation. Thus, resveratrol may reduce *S. aureus*-induced lung inflammation via the inhibition of c-Src/PDGFR/JNK1/2 and p38 MAPK activation.

Previous study proved that bacterial infections could enhance AP-1 activity [5]. AP-1 is composed of proteins belonging to the c-Fos, c-Jun, ATF, and JDP families, which can regulate gene expression in response to various stimuli [5]. AP-1 also has been shown to mediate various inflammatory genes expression, which can further promote the development of many inflammatory diseases. Moreover, we established that AP-1 plays a key role in mediating *S. aureus*-induced inflammatory responses. On the other hand, we observed that *S. aureus* induced c-Jun and ATF2 activation and in vivo binding of ATF2 or c-Jun to the VCAM-1 promoter via c-Src/PDGFR/JNK1/2 and p38 MAPK-dependent signaling in HPAEpiCs. These responses, induced by *S. aureus*, were inhibited by resveratrol.

**Figure 8:**
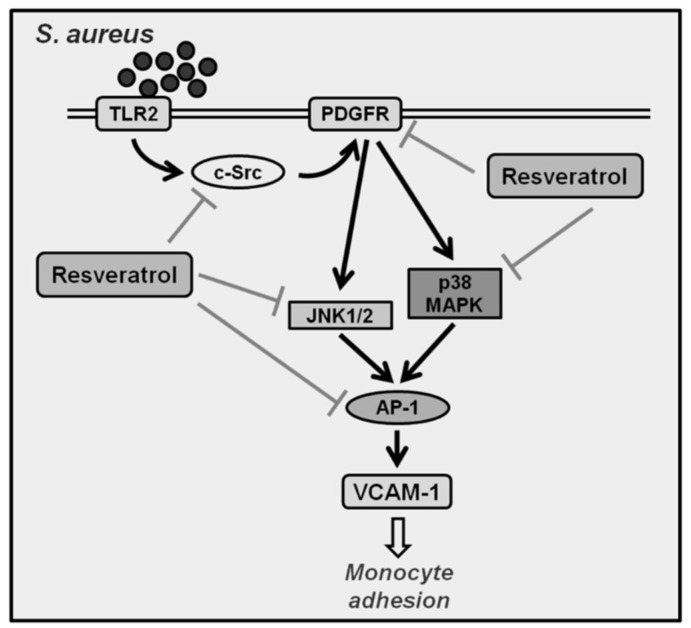
Schematic diagram illustrating the proposed signaling pathway involved in *S. aureus*-induced VCAM-1 expression in HPAEpiCs. *S. aureus* induces JNK1/2 and p38 MAPK activation via a c-Src/PDGFR pathway, which in turn initiates the activation of AP-1. Activated AP-1 is recruited to the promoter regions of VCAM-1, leading to an increase of VCAM-1 promoter activity and the expression of VCAM-1 mRNA and protein in HPAEpiCs. Moreover, resveratrol can reduce lung inflammation via the inhibition of VCAM-1 expression, monocyte adhesion, and the activation of c-Src, PDGFR, JNK1/2, p38 MAPK, and AP-1 induced by *S. aureus*.

In summary, as depicted in Figure 8, our results demonstrate that in HPAEpiCs, *S. aureus* induces JNK1/2 and p38 MAPK activation via a c-Src/PDGFR pathway, which in turn initiates the activation of AP-1. The activated AP-1 is recruited to the promoter regions of VCAM-1, leading to an increase of VCAM-1 promoter activity and the expression of VCAM-1 mRNA and protein in HPAEpiCs. Moreover, resveratrol can reduce lung inflammation via the inhibition of VCAM-1 expression, monocyte adhesion, and the activation of c-Src, PDGFR, JNK1/2, p38 MAPK, and AP-1 induced by *S. aureus*. 

## 4. Materials and Methods

### 4.1. Materials

PP1, AG1296, Tanshinone IIA, SP600125, and SB202190 were from Sigma (St. Louis, MO, USA). Anti-VCAM-1, anti-β-actin, anti-c-Src, anti-PDGFR, anti-p42, anti-p38, anti-JNK1, anti-c-Jun, and anti-ATF2 antibodies were obtained from Santa Cruz Biotechnology, Inc. (Santa Cruz, CA, USA). Anti-phospho-p38 MAPK, anti-phospho-JNK1/2, anti-phospho-p42/p44 MAPK, anti-phospho-c-Src, anti-phospho-PDGFR, anti-phospho-ATF2, and anti-phospho-c-Jun antibodies were obtained from Cell Signaling (Danver, MA, USA). Resveratrol was obtained from Sigma (St. Louis, MO, USA). Luciferase assay kit was obtained from Promega (Madison, WI, USA). BCECF/AM was obtained from Molecular Probes (Eugene, OR, USA). Enzymes and other chemicals were obtained from Sigma (St. Louis, MO, USA).

### 4.2. Cell Culture

Human pulmonary alveolar epithelial cells (HPAEpiCs) were purchased from ScienCell Research Lab (San Diego, CA, USA) and grown as previously described [7]. 

### 4.3. Preparation of S. aureus

*S. aureus* (strain 12598; a gift of J.C. Shu, Department of Medical Biotechnology and Laboratory Science, Chang Gung University, Tao-Yuan, Taiwan) was maintained in brain heart infusion broth (BHI; Sigma, St. Louis, MO, USA). The method of preparation of *S. aureus* was described in our previous study [2]. In each experiment, about 2 × 10^7^ bacteria, representing a bacteria-to-epithelial cell ratio of 20:1, were added to 1 mL of RPMI-1640 medium (Gibco BRL, Grand Island, NY, USA) in each well.

### 4.4. Animal Care and Experimental Procedures

Male Institute of Cancer Research (ICR) mice aged 6–8 weeks were obtained from the National Laboratory Animal Centre (Taipei, Taiwan) and handled according to National Institutes of Health (NIH) Guides for the Care and Use of Laboratory Animals. ICR mice were anesthetized with ethyl ether and placed individually on a board in a near vertical position; the tongues were withdrawn with a lined forceps. *S. aureus* (2 × 10^7^ CFU/mouse) was placed posterior in the throat and aspirated into lungs. Control mice were administrated BHI. Mice regained consciousness after 15 min. Mice were intraperitoneal given one dose of LY294002, SH-5, SP600125, U0126, Bay11-7082, or GR343 (2 mg/kg) or TLR2 or VCAM-1 neutralizing antibody (5 mg/kg) for 1 h or resveratrol (5 mg/kg) for 24 h prior to *S. aureus* (2 × 10^7^ CFU/mouse) treatment, and sacrificed after 24 h.

### 4.5. Isolation of BAL Fluid

Mice were intratracheally administered with *S. aureus* (2 × 10^7^ CFU/mouse) and sacrificed 24 h later. BAL fluid was obtained through a tracheal cannula using 1-mL aliquots of ice-cold phosphate buffered saline (PBS) medium. BAL fluid was centrifuged at 500× *g* at 4°C, and cell pellets were washed and re-suspended in PBS. Leukocyte count was determined by a hemocytometer.

### 4.6. Transient Transfection with siRNAs

Human siRNAs of c-Src (SASI_Hs01_00112905), PDGFR (SASI_Hs02_00341109), p42 (SASI_Hs01_00124656), JNK1 (SASI_Hs01_00010440), p38 (SASI_Hs01_00018464), c-Jun (SASI_Hs02_00333461), ATF2 (SASI_Hs01_00147372), and scrambled siRNAs were sourced from Sigma (St. Louis, MO, USA). Transient transfection of siRNAs (100 nM) was performed using a Lipofectamine^TM^ RNAiMAX reagent according to the manufacturer’s instructions.

### 4.7.Western Blot

We cultured HPAEpiCs in 6-well culture plates. After reaching confluence, HPAEpiCs were incubated with *S. aureus* for the indicated times at 37 °C. Western blot analysis methods were performed as previously described [2]. Finally, membranes were incubated with the anti-VCAM-1 antibody for one day and then incubated with the anti-mouse horseradish peroxidase antibody for 60 min. We used enhanced chemiluminescence (ECL) reagents to detect immunoreactive bands.

### 4.8. Real-Time RT-PCR 

We used TRIzol reagent to extract total RNA. We then reverse-transcribed mRNA into cDNA and analyzed it by real-time PCR, using SYBR Green PCR reagents (Applied Biosystems, Branchburg, NJ, USA) and primers specific for VCAM-1 and GAPDH mRNAs. Finally, VCAM-1 mRNA levels were determined by normalizing to levels of GAPDH expression.

### 4.9. Measurement of VCAM-1 Luciferase Activity

For construction of the VCAM-1-luc plasmid, human VCAM-1 promoter, a region spanning −1716 to −119 bp (kindly provided by W.C. Aird, Department of Molecular Medicine, Beth Israel Deaconess Medical Center, Boston, MA, USA) was cloned into a pGL3-basic vector (Promega, Madison, WI, USA). VCAM-1-luc activity was determined as previously described using a luciferase assay system (Promega, Madison, WI, USA) [3].

### 4.10. Adhesion Assay

HPAEpiCs were grown to confluence in 6-well plates, infected with *S. aureus* (2 × 10^7^ CFU/mL) for 24 h, and then adhesion assays were performed. Briefly, THP-1 cells (human acute monocytic leukemia cell line) were labeled with a fluorescent dye, 10 µM BCECF/AM, at 37 °C for 1 h in RPMI-1640 medium (Gibco BRL, Grand Island, NY, USA) and subsequently washed by centrifugation. Confluent HPAEpiCs in 6-well plates were incubated with THP-1 cells (2 × 10^6^ cells/mL) at 37 °C for 1 h. Non-adherent THP-1 cells were removed and plates were gently washed twice with PBS. The numbers of adherent THP-1 cells were determined by counting four fields per 200× high-power field well using a fluorescence microscope (Zeiss, Axiovert 200M). Experiments were performed in triplicate and repeated at least three times.

### 4.11. Chromatin Immunoprecipitation Assay

To observe the in vivo association of nuclear proteins with human VCAM-1 promoter, ChIP analysis was conducted as previously described [29] with some modifications. HPAEpiCs in 100-nm dishes were grown to confluence and serum-starved for 24 h. After infection with *S. aureus* (2 × 10^7^ CFU/mL), protein-DNA complexes were fixed by 1% formaldehyde in medium. The fixed cells were washed and lysed in SDS-lysis buffer (1% SDS, 5 mM EDTA, 1 mM phenylmethanesulfonylfluoride or phenylmethylsulfonyl fluorid (PMSF), 50 mM Tris-HCl, pH 8.1). The cell lysates were sonicated at 4 °C until the DNA length reached 200–1000 base pairs. The samples were centrifuged, and the soluble chromatin was precleared by incubation with sheared salmon sperm DNA-protein agarose A slurry (Upstate) for 30 min at 4 °C with rotation. Samples were centrifuged at 6000 rpm for 2 min and the supernatant was transferred to a new tube. The concentration of samples was quantified and balanced. One portion of samples was used as DNA input control, and the remains were subdivided into several portions, then incubated with an anti-ATF2 or anti-c-Jun antibody overnight at 4 °C. The immunoprecipitation complexes of antibody-protein-DNA were collected by using protein A-agarose beads for 1 h with rotation at 4 °C. Samples were successively washed with low-salt buffer (0.1% SDS, 1% Triton X-100, 2 mM EDTA, 20 mM Tris-HCl, pH 8.1, 150 mM NaCl), high-salt buffer (same as low-salt buffer but with 500 mM NaCl), LiCl buffer (0.25 M LiCl, 1% NP-40, 1% deoxycholate, 1 mM EDTA, 10 mM Tris-HCl, pH 8.1), and Tris-EDTA (pH 8.0), and then eluted with elution buffer (1% SDS, 100 mM NaHCO_3_). The cross-linking of protein-DNA complexes was reversed by incubation at 65 °C overnight. The DNA was extracted and re-suspended in H_2_O and subjected to PCR amplification with the forward primer 5’-AAATCAATTCACATGGCATA-3’ and the reverse primer 5’-AAGGGTCTTGTTGCAGAGG-3’, which were specifically designed from the VCAM-1 promoter region (−403 to −30). PCR products were analyzed on ethidium bromide-stained agarose gels.

### 4.12. Statistical Software and Analysis

We analyzed the data with the GraphPad Prism program (GraphPad, San Diego, CA, USA). Quantitative data were expressed as the mean ± S.E.M. and analyzed with one-way ANOVA followed with Tukey’s post-hoc test. We defined *p* < 0.05 as a significant difference.

## Abbreviations

*S. aureus*	*Staphylococcus aureus*
VCAM-1	Vascular cell adhesion molecule-1
SFKs	Src family kinases
HPAEpiCs	Human lung epithelial cells
MMP-9	Matrix metalloproteinase-9
EV71	Enterovirus 71
TLRs	Toll-like receptors
LPS	Lipopolysaccharide
